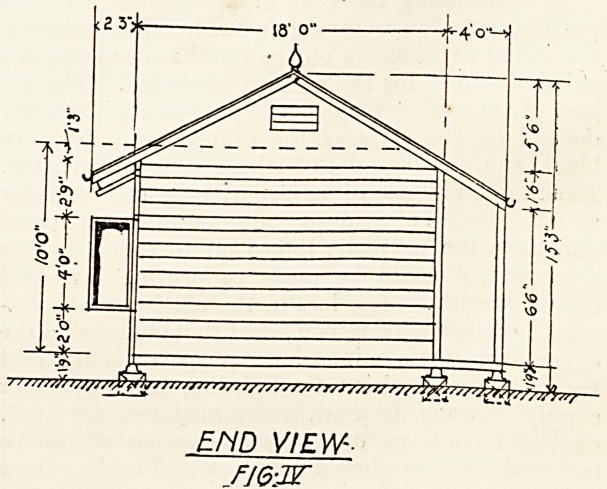# Economical Sanatorium Construction

**Published:** 1912-03-02

**Authors:** H. Hyslop Thomson

**Affiliations:** Medical Superintendent Liverpool Sanatorium, Frodsham, Cheshire.


### SANATORIUM CONSTRUCTION.

### II.—Economical Sanatorium Construction.

By H. HYSLOP THOMSON, M.D., D.P.H., Medical Superintendent Liverpool Sanatorium, Frodsham, Cheshire.

The type of sanatorium recommended for the treatment of tuberculosis amongst the industrial classes consists of a central administrative block of two floors containing the administrative quarters and accommodation for twenty patients. On each side of this block and connected with it by a covered way are two one-floor pavilions, each pavilion having accommodation for twenty patients. This gives accommodation for a hundred patients, which is regarded as the maximum number it is possible to treat efficiently in one institution.

### Points in Building.

The administrative block should be made of brick or reinforced concrete and contain a basement and two floors. The basement should contain two large drying-rooms through which patients enter and where they leave their boots, rugs, etc., before passing to their rooms. On the ground floor, in addition to the various rooms for administrative purposes, four single rooms should be reserved for the isolation of patients who have become seriously ill.

GROUND FLOOR PLAN
FIG I

In the type of sanatorium under consideration the night nurse is on duty in the administrative block into which all bells from patients' rooms ring so that she may be called when necessary. It is obvious, however, that for the efficient nursing of such conditions as severe hæmoptysis, pneumothorax, or tuberculous meningitis, isolation in a single room in the administrative block is essential, and it may be remarked in passing that such conditions will arise notwithstanding the most stringent preliminary examination. In addition to the single rooms, eight two-bedded rooms are required to provide accommodation for sixteen patients, and these rooms should be reserved chiefly for febrile cases. The importance of having accommodation for patients in the administrative block is also an economical question, as thereby the cost per head is reduced. To the rear of the main block the kitchen and dining hall should be situated, the later providing accommodation for one hundred patients. It is unnecessary to describe the other important details of the administrative department, as there is nothing specially distinctive connected with them. The style of the pavilion connected with the administrative block is important, as upon this the efficiency and economy of treatment chiefly depend. The type recommended in the present article, and particulars of which are shown in the accompanying plan and sketches, will be found to provide efficient treatment at a relatively low cost.

The pavilion consists of a one-floor building (fig. 1) with ten two-bedded rooms for patients, and a sitting-room and bedroom in the centre for a responsible nurse. Along the rear of the pavilion is a covered way 4 feet wide. Behind the centre of the pavilion and connected with it by a cross-ventilated passage is the sanitary and lavatory block. In front there should be a raised terrace for resting purposes in fine weather.

*FRONT VIEW OF ONE ROOM.*
*Fig. II.*

### The Question of Control.

For economical reasons it has been suggested that pavilions containing several patients in one room or ward should be erected, but the writer is strongly of opinion that in reactive cases of pulmonary tuberculosis the quieter the patient is kept the more speedily is the stage of injurious auto-inoculation cut short, and hence he recommends that not more than two patients should be together in one room. Moreover, where patients are accommodated in a block separated from the main building, it is absolutely necessary for the purposes of discipline and efficient treatment that a trustworthy nurse should be placed in authority over each pavilion. Did space permit, the writer could give some striking examples of the injurious results which have followed the absence of such controlling influence.

### Windows and Ventilation.

As shown in the accompanying sketches the size and arrangement of the windows secure efficient cross-ventilation. In front (see fig. 2) there are four French windows, which open outwards. Above these are four fanlights fixed to the overhanging eaves. This arrangement permits of the fanlights being kept open during the most inclement weather. In the rear wall of each room (see fig. 3) there is a large ordinary double sash window. This type of window is used, not only on account of consideration of space but because, as has been found from practical experience, the upper sash can be kept down to its full extent all the year round. Further cross-ventilation is secured by a ventilator above the

BACK VIEW SHEWING TWO ROOMS

FIG III

door; while in calm weather the door may be kept open by means of a spring-catch in the floor.

The size of the rooms gives approximately 1,000 cubic feet per patient, and any greater capacity than this is not required in view of the efficient cross-ventilation which is obtained. The material used in the construction of such a pavilion may be either wood, brick, or reinforced concrete. A serviceable and durable building may be constructed of wood preserved by steeping in creosote.

### A Lesson from Liverpool.

Bungalows partially made of this material have been used for treatment at the Liverpool Sanatorium for ten years, and have satisfactorily withstood exposure to severe weather conditions. All that is necessary to ensure preservation is a fresh application of the creosote preparation every three or four years. In erecting a wooden pavilion similar to that shown in the plan, the outer wall should be constructed of overlapping boards of creosoted wood, while the inner walls and ceiling should be made of closely-set tongued and grooved pine boarding. The boarding of the floor should also be tongued and grooved. To ensure protection from damp so necessary in a building constructed of wood, the pavilion should be raised at least one foot from the ground; concrete blocks with iron supports and wooden beams or girders being used for this purpose. In the sanitary block the floors must be of some impervious material, and the lower part of the walls should be tiled. The best roof for such a building, both from the point of view of durability and appearance, is undoubtedly red tile, but any impervious material may be used. Heating of the pavilion may be carried out by means of hot-water pipes or radiators, and these should be part of the general heating system for the whole institution. Heating, however, is not essential to efficient treatment, and may be dispensed with.

### Is Brick a Luxury?

While a pavilion constructed of wood as described above will prove an efficient structure for carrying out the principles of modern sanatorium treatment, the substitution of brick or reinforced concrete (for wood) is to be recommended when a slight increase in

END VIEW.

FIG IV

cost is not a matter of serious consideration. An efficient, durable, and picturesque pavilion for the accommodation of twenty patients on lines similar to the one described above may be constructed of brick roofed with red tile. In a one-floor pavilion of this kind constructed of brick several points call for special attention. In place of brick foundations with damp-proof course the pavilion should rest on brick arches, or steel girders supported by iron rests and concrete blocks, so as to be raised above the ground. The inside walls should be made of the best hydraulic plaster, and the boarding of the floors should be tongued and grooved and closely set. Special attention must be paid to the rounding of angles and corners, and to the junction between plaster work and wood work.

The floor of the covered-way which runs along the rear of the pavilion should be made of closely-set boarding, previously steeped in creosote. Reinforced concrete may also be used for the construction of such a pavilion, but it lacks the pleasing appearance of good brick. The relative cost of pavilions constructed of wood, brick, and concrete is given below. One source of expenditure in building a sanatorium is the provision of special shelters for the accommodation of patients during the hours of rest. This point is considered here for the reason that in the scheme under consideration provision is made in the rooms in the pavilion for rest-chairs, which can be carried out of doors in fine weather, so that special shelters are unnecessary.

### No Need for Shelters.

A suitable shelter for two patients will cost, according to its type and material, from £5 to £20, but the experience of the writer is that under the pavilion scheme the provision of such shelters is not necessary for efficient treatment. A special rest-chair made of creosoted wood and canvas, specially prepared to resist the effects of moisture, and which is sufficiently light to be easily carried by a nurse or by a patient free from fever, can be provided at the cost of sixteen shillings. Lastly, the pavilion should be well protected from cold winds, and this is best secured by means of pine trees planted close together.

### Cost of Scheme.

In considering the cost of a sanatorium of the pavilion type to accommodate one hundred patients, the initial expenditure on the purchase or lease of a suitable site is for the present excluded. The extent of the outlay for building purposes will greatly depend on the style of the central administrative block and the material of which it is constructed. Existing buildings of sufficient size and suitably situated may be used for administrative purposes and have the pavilions linked on to them. This arrangement could be made to provide increased economy without any loss in the efficiency of treatment. It has to be remembered that included in the expenditure for administrative purposes is the cost for adequate provision for lighting, heating, water supply, sewage disposal, accommodation for staff, etc., and while no unnecessary expense should be incurred economy should not be carried to the extent of impairing the efficiency of treatment.

An administrative block, with accommodation for twenty patients, constructed of brick and roofed with tile, would cost probably not less than £10,000. If, however, reinforced concrete, or wood and corrugated iron be used, the requirements of efficient treatment could be met at a much lower figure. The risk of fire, however, is a serious objection to the employment of wood in a large building of more than one story.

### Cost per Pavilion.

The maximum cost of the pavilions to accommodate twenty patients, if built of brick and roofed with tile, may be put at £2,000 each. This price represents first-class workmanship, and includes the following:—

£40 for heating boiler and circulating pipes.
£5 for each bath.
£5 for each douche.
£3 for each closet.
£2 for each lavatory basin.

The above quotation makes allowance for difficulty in obtaining bricks, and as it is a first-class specification, a lower one could no doubt easily be obtained.

If creosoted wood with tile roofs be used instead of brick the maximum cost for first-class workmanship would be about £1,700 for each pavilion.

With regard to furniture, which must be included in estimating the cost per bed, £17 is allowed for each two-bedded room. This includes two beds, two rest-chairs, two lockers, two chairs, and a double wardrobe with separate doors and sides perforated for ventilation.

### Cost per Bed.

From the figures given it will be seen that over and above that provided in the administrative block accommodation can be obtained in suitable pavilions equipped and furnished at a cost of less tha £100 per bed. By means of an administrative block constructed of reinforced concrete and connected with one-story pavilions made of wood, accommodation for one hundred patients could be provided at a cost of less than £200 per bed. If brick be used for the construction of the administrative block and pavilions, a sanatorium on this principle to accommodate a hundred patients could be erected at a cost not exceeding £250 per bed. Lastly, with regard to maintenance, it is only necessary to add that, when dealing with one hundred patients, efficient treatment can be carried out at a cost not exceeding 25s. per head per week.

In concluding, the writer has to express his thanks to Mr. Glover, of Holt and Glover, architects, Liverpool, who has kindly supplied the necessary particulars as to the cost of the pavilions, and to Mr. Plant, engineer to the Liverpool Sanatorium, who has kindly prepared the plan and sketches.

## Figures and Tables

**FIG I f1:**
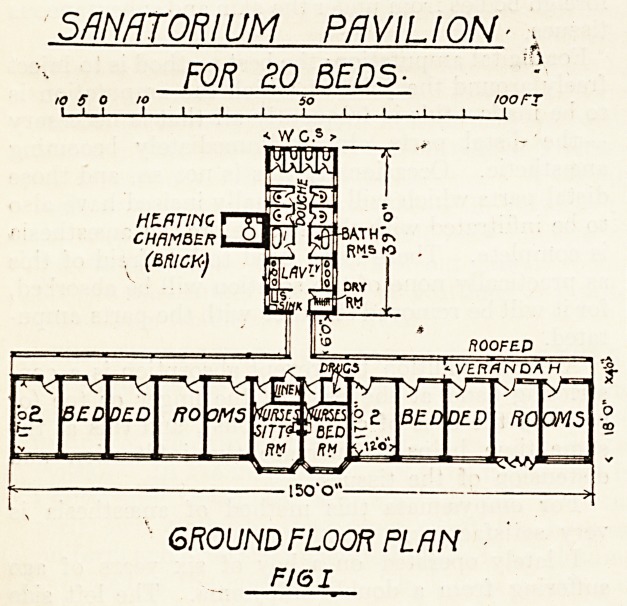


**Fig. II. f2:**
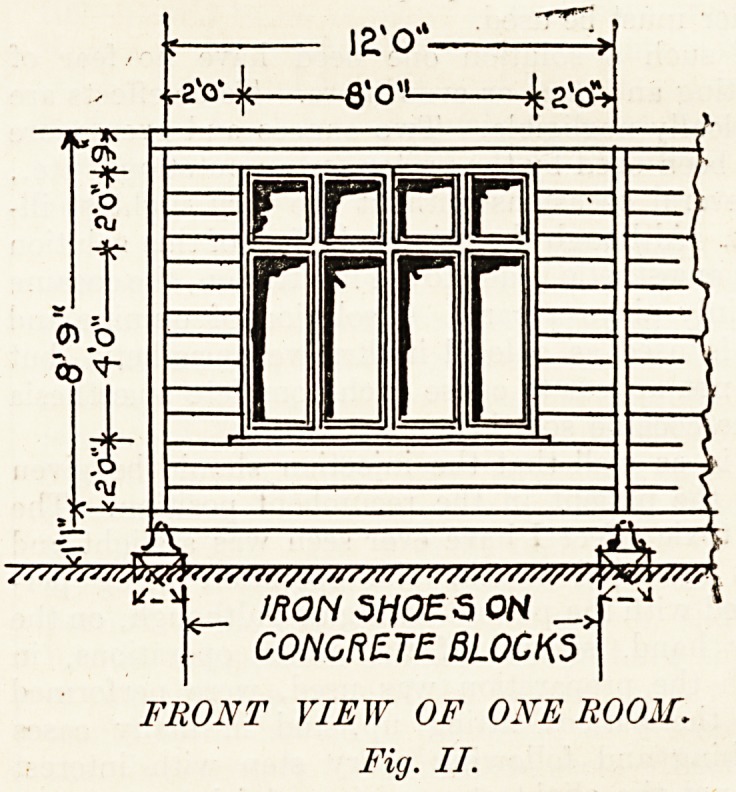


**FIG III f3:**
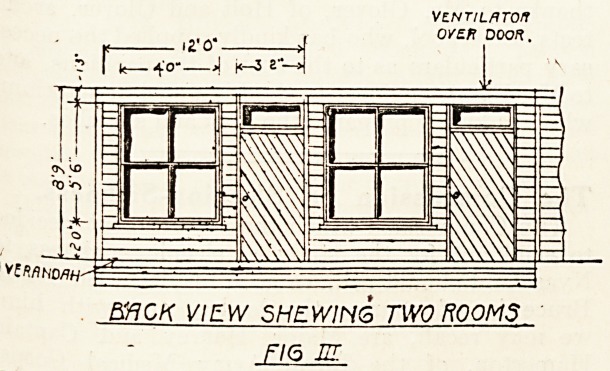


**FIG IV f4:**